# Effects of virtual reality on pain, anxiety, and delirium in pediatric surgery: a systematic review and meta-analysis

**DOI:** 10.1007/s00383-025-06084-w

**Published:** 2025-06-10

**Authors:** Seçil Taylan, Kızbes Meral Kılıç, İlknur Özkan

**Affiliations:** 1 Kumluca Faculty of Health Sciences, Surgical Nursing Department, Akdeniz University, Kumluca, Antalya Turkey; 2 Kumluca Faculty of Health Sciences, Child Development Department, Akdeniz University, Kumluca, Antalya Turkey; 3 Kumluca Faculty of Health Sciences, Internal Medicine Nursing Department, Akdeniz University, Kumluca, Antalya Turkey

**Keywords:** Virtual reality, Nursing, Pediatrics, Meta-analyses

## Abstract

**Aim:**

To examine the effects of virtual reality on preoperative, intraoperative and postoperative patient outcomes in pediatric surgical patients, providing a comprehensive assessment of its potential benefits in perioperative care management.

**Methods:**

This research employed a systematic review and meta-analysis methodology. The databases MEDLINE, Complementary Index, Academic Search Ultimate, CINAHL Complete, Directory of Open Access Journals, and Supplemental Index were systematically searched using keywords adapted to each database. To evaluate the quality of randomized controlled trials (RCTs), the Cochrane Risk of Bias Tool (RoB 2) was utilized. The data analysis was conducted using Comprehensive Meta-Analysis (CMA) software version 3. Meta-analyses were performed on studies that provided continuous statistical data suitable for estimating overall effects.

**Results:**

Virtual reality significantly reduced preoperative and intraoperative anxiety to a moderate degree (p < 0.001 and p = 0.002). However, its effects on postoperative pain (p = 0.071) and delirium (p = 0.307) were not statistically significant.

**Conclusion:**

Virtual reality is an effective intervention for reducing anxiety in pediatric surgical patients. However, considering the heterogeneity and methodological differences among the included studies, further randomized controlled trials (RCTs) are necessary to strengthen these findings.

## Introduction

The management of surgical processes has a significant impact on patient outcomes [1]. Compared to adults, children are more vulnerable psychologically, emotionally, and physiologically, making the management of surgical processes more challenging, complex, and requiring greater care [2]. Pediatric patients may face various psychological and physiological difficulties at different stages of surgical procedures [3]. Before surgical intervention, patients may experience anxiety about pain or the possibility of experiencing pain during the procedure [4]. Additionally, the fear of separation from family and anxiety about unfamiliar situations can be significant stress factors[4, 5]. Postoperatively, patients may encounter undesirable outcomes such as pain, fear, inappropriate behaviors, and delirium [6, 7]. These factors can have a considerable impact on the management of the surgical process and the recovery period [6]. Proper management of pediatric surgical patients can improve perioperative patient outcomes [8]. Timely and appropriate interventions can reduce the levels of stress, anxiety, and pain experienced by children before, during, and after surgery, thereby accelerating the recovery process [9]. Moreover, such measures can facilitate their adaptation to surgical procedures, enhance overall patient satisfaction, and minimize the risk of complications [8]. Additionally, incorporating play elements (gamification) can support children's learning processes and reduce sedation requirements, thereby lowering associated risks [3].

One of the non-pharmacological methods used in the perioperative care management of pediatric patients is virtual reality (VR) applications [4]. VR can be classified into two main types: immersive and non-immersive. Non-immersive VR reproduces three-dimensional environments using conventional graphical devices, whereas immersive VR fully immerses the user in a simulated world through headsets, visors, and motion tracking systems. This type of cognitive and sensory immersion presents an innovative non-pharmacological therapeutic alternative that can have a positive impact on perioperative patient outcomes [3]. VR has emerged as an effective intervention tool in reducing anxiety, pain, and distress experienced by children before and after surgical procedures [10]. Randomized studies have shown that VR applications facilitate children's adaptation to surgical interventions by helping to reduce pain, delirium, fear, and inappropriate patient behaviors while accelerating the recovery process [11, 12]. Recent meta-analysis studies have confirmed that VR is effective in reducing anxiety and pain in children undergoing medical procedures [10, 13–15].

The perioperative process is composed of three primary phases: preoperative, intraoperative, and postoperative. Each phase differs in its distinct dynamics and operational characteristics [16]. These three interdependent phases influence one another sequentially and have the potential to shape patient outcomes. The optimization of each phase can significantly enhance the overall efficacy of the perioperative process, patient outcomes, and recovery trajectories [17].A recent meta-analysis has demonstrated that VR interventions effectively reduce surgery-related anxiety in pediatric patients [3].

There is a clear gap in the literature regarding studies that systematically evaluate the effects of virtual reality (VR) interventions in the preoperative, intraoperative, and postoperative phases among pediatric surgical patients. These three phases represent clinically and dynamically distinct stages, yet the extent to which VR is effective in each phase, the mechanisms through which it produces its effects, and how these effects influence patient outcomes have not been fully clarified. Addressing this gap will contribute to the development of evidence-based clinical decisions regarding when, how, and in which phase VR should be applied in pediatric surgical care. This systematic review and meta-analysis aims to evaluate the effects of virtual reality in each perioperative phase separately, thereby providing a scientific foundation for the development of targeted intervention strategies to enhance the effectiveness of pediatric surgical care.

## Method

### Study design

This research employed a systematic review and meta-analysis methodology, adhering to the principles set forth in the Preferred Reporting Items for Systematic Reviews and Meta-Analyses (PRISMA) guidelines [18]. To ensure methodological rigor and transparency, the study protocol was registered with the International Prospective Register of Systematic Reviews (PROSPERO) under the registration number CRD420250650777.

### Inclusion and exclusion criteria

The research questions were developed using the Participants, Intervention, Comparator, and Outcome (PICO) framework [19]. In this study, the intervention (I) involved virtual reality applications, while a control group (C) was included in all studies to compare VR interventions with standard hospital care. The outcomes (O) assessed were anxiety, pain, and delirium in pediatric surgical patients.Based on this framework, the study aimed to address the following research questions:

- Do virtual reality interventions effectively reduce anxiety, pain, and delirium in pediatric surgical patients?
- If effective, what is the impact of these interventions on patient outcomes?

Studies were excluded if they met at least one of the following criteria: (1) published in languages other than English; (2) abstracts/posters; (3) studies without a control group or studies; (4) published in non-peer-reviewed journals; (5) theses, letters, committee reports, conference proceedings, short articles, and expert opinions; and (6) systematic reviews and meta-analyses.

### Search strategy

**Fig. 1:**
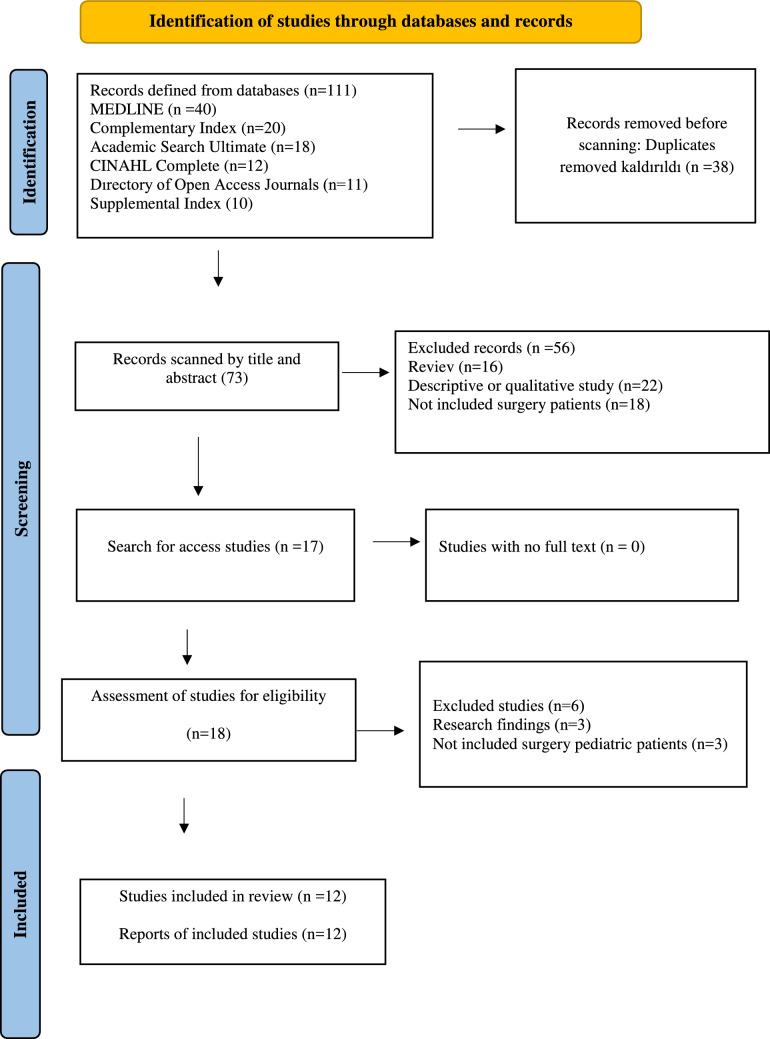
PRISMA flow diagram

The databases MEDLINE, Complementary Index, Academic Search Ultimate, CINAHL Complete, Directory of Open Access Journals, and Supplemental Index were systematically searched using keywords adapted to each database. No time restrictions were imposed, and all studies published up to December 2024 were included. The search strategy used the following terms: (virtual reality OR VR OR augmented reality) AND (pediatric OR child OR children OR infant OR adolescent) AND (surgery OR operation OR surgical* OR operative) AND (randomized controlled trials OR RCT OR randomised control trials) (Fig. 1).

### Selection of studies

A three-phase selection process was employed to manage the literature search records, utilizing EndNote X8 reference management software. The selection was independently conducted by the authors (ST, İÖ, and MKK), who later convened to discuss and reach a consensus. In the first phase, article titles were reviewed, and those requiring further clarification were moved to the next stage for additional evaluation. During the second phase, the abstracts of the shortlisted articles were examined in detail. In the final phase, full-text articles were carefully assessed to ensure they met the predefined inclusion criteria before inclusion in the study.

### Data extraction

During the data extraction phase, a standardized data extraction form consisting of nine predefined items was used. This form had been previously tested to ensure consistency and reliability. The extracted data included the following information: (i) author names, (ii) publication year, (iii) country where the study was conducted, (iv) study duration, (v) sample size, (vi) type of intervention, (vii) mean scores for post-intervention measures, (viii) standard deviations for post-intervention measures, and (ix) a description of the intervention.

### Quality assessment

**Fig. 2:**
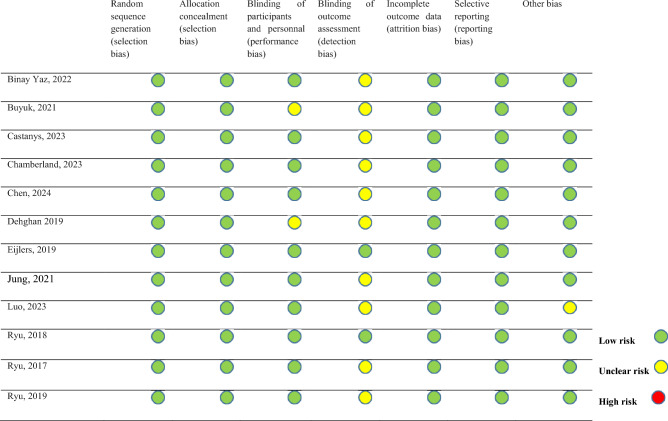
Risk of bias summary (ROB2)

Two authors (ST and İÖ) independently assessed the quality of the studies, with a particular focus on methodological bias, and later reached a consensus {Sterne, 2019 #59}. To evaluate the quality of randomized controlled trials (RCTs), the Cochrane Risk of Bias Tool (RoB 2) was utilized (Fig. 2).

### Data analysis

The data analysis was conducted using Comprehensive Meta-Analysis (CMA) software version 3. Meta-analyses were performed on studies that provided continuous statistical data suitable for estimating overall effects. Hedges' g was used to determine effect sizes, which were classified as small (0.2), moderate (0.2–0.8), and large (≥ 0.8) [20].

To assess heterogeneity, Chi-square (χ^2^) tests and I^2^ statistics were utilized. A fixed-effects model was applied for studies with I^2^ ≤ 50% and p > 0.1, while a random-effects model was used when I^2^ > 50% and p > 0.1. The degree of heterogeneity was categorized as low (I^2^ = 25%), moderate (I^2^ = 50%), and high (I^2^ = 75%). Since variations in methodology and participant characteristics were expected, the random-effects model was preferred for estimating effect sizes. The Tau^2^ statistic was also employed to measure variance and heterogeneity, where Tau^2^ = 0.000 indicated homogeneity and justified the use of a fixed-effects model. Potential publication bias was assessed using a funnel plot, alongside Egger’s and Begg & Mazumdar tests. Effect sizes were presented with 95% confidence intervals (CIs), and statistical significance was set at p < 0.05.

### Ethical considerations

This study followed ethical guidelines for systematic reviews and meta-analyses. Since no direct human participants were involved, ethical approval was not required. However, all included studies were reviewed for prior ethics committee approval. The research adhered to PRISMA guidelines, ensuring transparency and methodological integrity.

## Results

### Search outcome

A search conducted across six electronic databases identified 111 records. After removing duplicates using EndNote, 73 studies remained for screening. Titles and abstracts were reviewed, leading to the exclusion of 56 studies. The full texts of 18 studies were assessed for eligibility, and 12 studies meeting the inclusion criteria were included in the meta-analysis (Fig. 1).

### Quality assessment

#### Risk of bias assessment

The risk of bias assessment is presented in Fig. 2. All included studies reported randomization, and the method of randomization was explicitly described in each study. Therefore, these studies were classified as low risk in the first two phases of the quality assessment tool. Regarding blinding, two studies did not mention blinding procedures [21, 22], while two studies specifically reported blinding of outcome assessment [23, 24]. In terms of other potential sources of bias, one study did not disclose financial support details [25], leading to its classification as unclear risk. The remaining studies reported no commercial funding, and thus were considered low risk (Fig. 2).

### Characteristics of ıncluded studies and ıntervention strategies

An analysis of the included studies and their intervention strategies revealed that all studies were conducted between 2017 and 2024, representing recent research in the field (Table 1). Three studies implemented interventions in three different groups. In one study, the Educational Animation Group (EAG) and Documentary Group (DG) were compared in addition to the control group [26]. Another study evaluated Biophilic-VR (BVR) and Indoor-VR (IVR) groups [22]. In a third study, a tablet-based intervention was applied alongside the control group [27].

**Table 1:** Characteristics of the studies

First author Study year Country	Participiants	Groups (n)	Aim	VR Equipment	Measures (Scale)	Intervention	Outcome
	Surgical procedure						
Binay Yaz 2022 Turkey	Children aged 6 to 12 years old	VR-EAG (44) VR-DG (44) CG (44)	To investigate the effects of watching an educational animated movie on fear and pain in children aged 6 to 12 years old	Unknown	Pain (W-BFPRS) Fear (CFS)	Depending on the study group, children watched a 3–4 min educational animated film or VR documentary. Afterward, children completed the CFS, and nurses completed the W-BFPRS when the children returned to their room and one hour postoperatively	The educational animated movie was found to be an effective method in reducing preoperative fear and postoperative pain
	Unknown						
Buyuk 2021 Turkey	5–10-years-old boys	VR (n = 40) CG(n = 38)	To examine the effects of using virtual reality (VR) intervention before circumcision on the pre-and postoperative anxiety and fear levels and postoperative pain symptoms in children	A white “VR BOX 3.000” with VR glasses compatible with iOS and Android operating systems and 4–6 inch screen smartphones was used. The weight of the VR glasses is 0.414 kg. Soundproof headphones are used to provide immersive VR	Pain (W-BFPRS) Fear (CFS) Anxiety (CAMS)	Before the surgery, the children were allowed to watch the VR program of their choice, the average duration of which was 4.5 min, using the VR glasses. Preoperative and postoperative anxiety and fear and postoperative pain were measured	Distracting children using a VR intervention before circumcision decreased their anxiety and fear both before and after the surgery, and it was found that the pain symptoms were lower in the postoperative period
	Circumcision						
Castanys 2023 Spain	Children (ASA I-II) aged 4–12	VR (n = 61) CG(n = 64)	*T*o compare the level of preoperative anxiety in children after standard preparation plus a virtual tour of the operating room vs. standard preparation alone	The VT Kit included virtual reality goggles (made from cardboard and plastic lenses, using the parent's mobile phone as a screen) and instructions for streaming the 360° video through the Nixi for Children web app	Anxiety (YPAS) Delirium (PAEDS) Behavior (PHBQ) Induction Compliance (ICC) Anxiety (STAI) Anxiety (VAS)	It starts with Nixi, an animated 3D character who must undergo surgery and a professional actress who acts as an anesthesiologist and accompanies children along the 3-min 48-s virtual tour, including the reception, OR, and post-anesthetic recovery room	Virtual tours for perioperative patients may reduce perioperative anxiety and improve satisfaction
	Outpatient surgery						
	Surgical procedure						
Chamberland, 2023 Canada	Children and adolescents aged 5 -17 years old	VR (n = 37) CG (n = 64)	To determine whether augmented reality could reduce preoperative anxiety in pediatric patients undergoing elective day surgeries	AR intervention. through Microsoft HoloLens 2 glasses. The animation follows the story of a character called "Constellation", who is a celestial body that lives among the stars and travels through the universe	Anxiety (YPAS)	CG patients received standard care. Standard care included attention and comforting and supportive words during the procedures. VR in addition to standard procedure, patients in the intervention group received AR intervention	Use of augmented reality before induction of general anesthesia reduced anxiety in children and adolescents
	Undergoing elective day surgeries						
Chen, 2024 China	Children aged 4–7 years old	VR (n = 30) TG (n = 30) CG (n = 30)	To evaluate whether an immersive VR distraction intervention or a combination of parental presence and video distraction is an effective strategy for preschool-aged children undergoing general anesthesia and elective surgery	Individualized intervention using VR technology (PICO 4 Vision Pro). During preoperative handover and anesthesia induction, the child watched a VR video, which resumed post-surgery until PACU discharge, without parents present for group VR	Anxiety (PSAS) Pain (FLACC) Delirium (PAEDS)	Group V underwent a preoperative consultation aimed at identifying the child's preferred animations for the immersive	Including VR technology in the perioperative period has positively affected postoperative outcomes
	Elective surgery						
Dehghan 2019 Iran	Children aged 6–12 years old	VR (n = 10) CG (n = 10)	To investigate the effect of virtual reality technology on pre-operative anxiety in children	After selecting study samples, participants were divided into two interventional groups and two control groups randomly and the pretest was executed in two groups (interventional 1 and control 1) out of four groups In the interventional group, patients experienced a virtual reality simulation of entering the operating room using eyeglasses and headphones, while in the control group, parents comforted their children with touch and caresses	Anxiety (YPAS)	The interventional group had a 5-min exposure to the operating room using virtual reality technology, but the control group did not receive virtual reality exposure	The medical treatment using virtual reality technology, as well as distraction and drowning in the virtual reality, reduced pre-operative anxiety in children
	Abdominal surgery						
Eijlers, 2019 The Netherlands	Children aged 4 to 12 years old	VR (n = 94) CG (n = 97)	To investigate if virtual reality exposure (VRE) as a preparation tool for elective day care surgery in children is associated with lower levels of anxiety, pain and emergence delirium compared with a control group receiving care as usual (CAU)	HTC Vive visor, monitor PC. Video virtual reality of the operating room in two versions (4–7 years) and (8–12 years)	Anxiety (mYPAS) Anxiety (VAS) Anxiety (STAI) Pain ((FPS-r) Pain (FLACC) Pain (PPPM) Deliryum (PAED)	Their anesthesiologist during the preoperative screening consultation to watch the informative online movie of the Erasmus MC-Sophia Children’s Hospital about general anesthesia prior to surgery	In children undergoing elective day care surgery, VRE did not have a beneficial effect on anxiety, pain, emergence delirium or parental anxiety
	Elective Pediatric Day Care Surgery						
Jung 2021 USA	Children aged 5 to 12 years old	VR (n = 33) CG (n = 37)	To determine whether immersive audiovisual distraction with a VR headset during induction of general anesthesia (GA) in pediatric patients reduced preoperative anxiety	Samsung Gear VR headset (Samsung Electronics, Suwon, South Korea) that displayed a preselected, interactive game (Mighty Immersion, Palo Alto, CA)	Anxiety = mYPAS	VR group patients received audiovisual distraction via a VR headset during induction, while the control group received none. The primary outcome, mYPAS, measured anxiety at three points: pre-randomization in the holding area, on OR entry, and during induction	This study demonstrates a reduction in pediatric preoperative anxiety with the use of VR
	Elective Surgery						
Luo, 2023 China	Children aged 7–18 years old	BVR (n = 34) IVR (n = 36) CG (n = 36)	To evaluate the effects of the biophilic virtual reality (BVR) method on children’s pain and anxiety undergoing circumcision	In the BVR group, children experienced rural nature scenes with a blue sky, clouds, trees, water, birds, and soft music, while in the IVR group, they saw an apartment room with large windows and furniture, including a TV	Anxiety (VAS) Faces Pain Scale-Revised (FPS-R) Heart Rate Pain Index (PI) Anxiety (STAI) Anxiety Index (AI)	The BVR and IVR groups wore VR glasses from OR entry until surgery ended, while the control group did not. A blinded researcher conducted assessments at T0 (holding area) and T1-T6 (surgery stages) post-randomization. VR glasses were removed after surgery	Intraoperative VR may be an effective noninvasive modality for reducing pain and anxiety during circumcision. PI and AI might be used to assess subjective pain and anxiety in patients
	Circumcision						
Ryu 2018 Korea	Children aged 6 to 15 years old	VR (n = 34) CG (n = 35)	To evaluate whether gamification of the preoperative process through virtual reality (VR) games can reduce preoperative anxiety in children	VR game producing company (JSC GAMES, Seoul, Korea)	Anxiety = mYPAS Anxiety = ICC Behavior = PBRS	Children in the control group received conventional education regarding the preoperative process, whereas those in the gamification group played a 5 min VR game experiencing the preoperative experience	VR experience of the preoperative process could reduce preoperative anxiety and improve compliance during anesthetic induction in children undergoing elective surgery and general anesthesia
	Elective Surgery						
Ryu 2017 Korea	Children aged 4 to 10 years old	VR (n = 34) CG(n = 35)	To determine whether a preoperative VR tour could reduce preoperative anxiety in children	The VR tour was provided as a 360∘ movie that introduced and further explained the perioperative preparation process. The 4-min video was produced in collaboration with IONIX (Seongnam, Korea) and a VR producing company (The VR, Seoul, Korea)	Anxiety = mYPAS Anxiety = ICC Behavior = PBRS	The control group received conventional information regarding anaesthesia and surgery. The VR group watched a 4-min video showing Pororo, the famous little penguin, visiting the operating theatre and explaining what is in it	This preoperative VR tour of the operating theatre was effective in alleviating preoperative anxiety and increasing compliance during induction of anaesthesia in children undergoing elective surgery
	Elective Surgery						
Ryu 2019 Korea	Children aged 4 to 10 years old	VR (n = 41) CG(n = 39)	To determine whether a preoperative immersive virtual reality tour reduces emergence delirium by decreasing preoperative anxiety in children undergoing general anesthesia	The VR video, created by IONIX and The VR Co., featured Pororo The Little Penguin characters in partnership with Seoul National University Bundang Hospital. Filmed in the hospital with modified GoPro Hero4® cameras, it was later developed into a mobile app for Galaxy S6® phones with VR Gear® headsets	Delirium = PAED Anxiety = mYPAS Behavior = PHBQ‐AS	The control group received conventional education regarding the perioperative process. The virtual reality group watched a 4-min virtual reality video showing the operating theater and explaining the perioperative process	Preoperative immersive virtual reality tour of the operating theater did not reduce the incidence and severity of emergence delirium, although it was effective in alleviating preoperative anxiety in children
	Elective Surgery						

*CFS* Children’s fear scale, *W-BFPR*S Wong-baker faces pain rating scale, *CAMS* Children’s anxiety meter scale, *PAEDS* Pediatric anesthesia emergence delirium scale, *PHBQ* Post hospitalization behavior questionnaire, *ICC* Induction compliance checklist, compliance, *STAI* State–Trait Anxiety Inventory, *VAS* Anxiety visual analog scale, *CEMS* Emotional manifestation scale, *CENS* Childhood emotional manifestation scale, *AI* Anxiety ındex, *FPS-R* Faces pain scale-revised, *PI* Pain ındex; *PSAS* Parental separation anxiety scale, *FLACC* Faces, legs, activity, cry, consolability, *FPS* Faces pain scale, *VSAS* Venham situational anxiety scale, *PPPM* Parents’ postoperative pain measure, *FAS* Facial affective scale, *PBRS* Procedural behavior rating scale, *PHBQ‐AS* Behavior questionnaire for ambulatory surgery

The included studies were conducted in the following countries: South Korea (n = 3) [24, 28, 29], Turkey (n = 2) [26, 30], China (n = 2) [22, 31], Spain (n = 1) [25], Canada (n = 1) [32], Iran (n = 1) [21], the Netherlands (n = 1) [23], and the United States (n = 1) [33].The total number of participants across all studies was 1,101 (VR group: n = 572, Control group: n = 529), with sample sizes ranging from 10 to 97 per study group.

The meta-analysis evaluated the effects of VR interventions in the perioperative period, with participants undergoing various surgical procedures. One study did not specify the type of surgery performed [26]. In two studies, VR was used in circumcision surgeries [22, 30]. Another study focused on outpatient surgical procedures [25], while one study assessed its application in abdominal surgery [21]. The remaining seven studies involved elective surgical procedures, covering a broad range of planned, non-emergency surgeries [24, 27–29, 32–34].

In the meta-analysis, the impact of VR on preoperative anxiety was assessed in 10 studies [21–25, 28–30, 32, 33]. Additionally, the effect of VR on heart rate and pain in the preoperative phase was examined in one study [22].

In this meta-analysis, the effects of virtual reality (VR) interventions during the intraoperative period were evaluated across multiple studies. The impact of VR on anxiety was assessed in six studies [22–24, 27, 29, 33]. In five of these, children underwent general anesthesia, and anxiety levels were typically measured while the children were still conscious—either immediately before or during anesthesia induction in the operating room setting [23, 24, 27, 29, 33]. The effect of VR on pain was examined in two studies [22, 31], while its influence on induction compliance and heart rate was each explored in one study [22, 25]. Additionally, delirium was evaluated in one study, and behavioral responses were investigated in two studies [24, 29].

In the meta-analysis, the impact of VR in the postoperative period was assessed across multiple studies. The effect of VR on pain was examined in three studies [23, 26, 30], while its impact on delirium was analyzed in three studies [23, 25, 28]. The influence of VR on anxiety was evaluated in two studies [23, 30], and its effect on behavioral responses was investigated in one study [28]

In the meta-analysis, the impact of VR after hospital discharge was assessed in one study for its effects on pain and anxiety [23]. Additionally, the effect of VR on delirium on postoperative day 14 was evaluated in one study [28].

### Effect of ınterventions

Meta-analysis combines statistically comparable continuous data to estimate overall effects. In this study, the approach recommended in the literature, which suggests conducting a meta-analysis when at least three or more similar studies provide combinable statistical data, was followed [35]. To assess the impact of VR on patient outcomes during the surgical process, this meta-analysis synthesized findings related to preoperative, intraoperative, and postoperative effects of VR interventions**.**

**Fig. 3:**
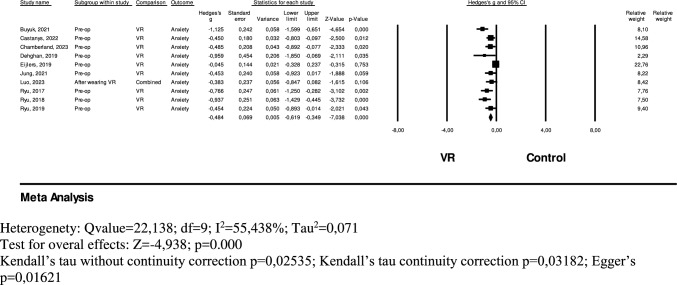
Forest plot of the effect of VR on pre-operative anxiety

A total of 454 pediatric surgical patients in the VR group and 455 in the control group were included in the analysis to evaluate the effect of VR on preoperative anxiety. The model exhibited high heterogeneity (Q = 22.138, df = 9, I^2^ = 55.438%, Tau^2^ = 0.071), leading to the application of a random-effects model in the meta-analysis. Results indicated that VR interventions significantly reduced preoperative anxiety to a moderate degree (Hedges' g = − 0.484, 95% CI = − 0.619 to − 0.349, p < 0.001). However, publication bias was detected based on Kendall’s tau without continuity correction (p = 0.02535), Kendall’s tau with continuity correction (p = 0.03182), and Egger’s test (p = 0.01621) (Fig. 3).

**Fig. 4:**
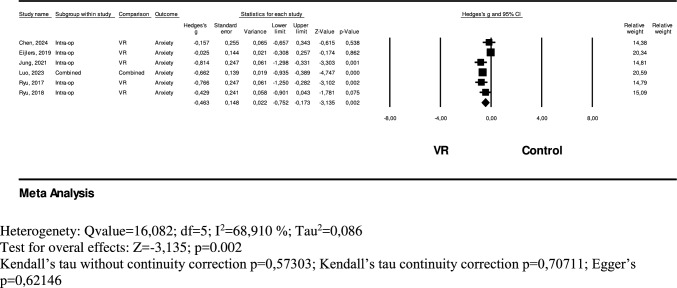
Forest plot of the effect of VR on intra-operative anxiety

A total of 295 pediatric surgical patients in the VR group and 179 in the control group were included in the analysis to evaluate the effect of VR on intraoperative anxiety. The model exhibited high heterogeneity (Q = 16.082, df = 5, I^2^ = 68.910%, Tau^2^ = 0.086), leading to the application of a random-effects model in the meta-analysis. Results indicated that VR interventions significantly reduced intraoperative anxiety to a moderate degree (Hedges' g = − 0.463, 95% CI = − 0.752 to − 0.173, p = 0.002). However, publication bias was not detected, as indicated by Kendall’s tau without continuity correction (p = 0.57303), Kendall’s tau with continuity correction (p = 0.70711), and Egger’s test (p = 0.62146) (Fig. 4).

**Fig. 5:**
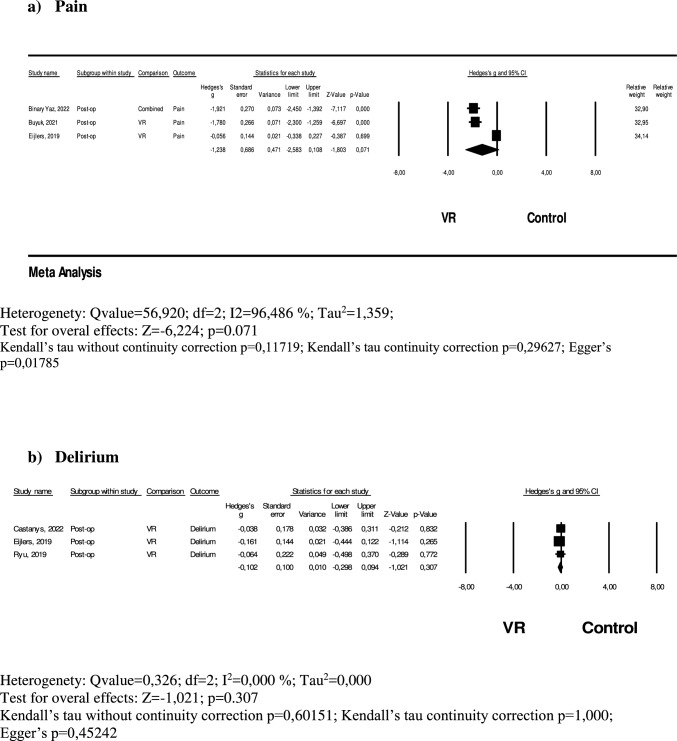
**a** Forest plot of the effect of VR on post-operative pain. **b** Forest plot of the effect of VR on post-operative process

A total of 222 pediatric surgical patients in the VR group and 270 in the control group were included in the analysis to evaluate the effect of VR on postoperative pain [23, 26, 30]. A random-effects model was used in the meta-analysis due to the model exhibiting very high heterogeneity (Q = 56.920, df = 2, I^2^ = 96.5%, Tau^2^ = 1.359).The meta-analysis indicated that VR interventions reduced postoperative pain to a high degree compared to the control group; however, this effect was not statistically significant (Hedges' g = − 1.238, 95% CI = − 2.583 to 0.108, p = 0.071). Publication bias was not detected according to Kendall’s tau without continuity correction (p = 0.11719) and with continuity correction (p = 0.29627), whereas Egger’s test (p = 0.01785) suggested the presence of publication bias (Fig. 5a).

A total of 196 pediatric surgical patients in the VR group and 200 in the control group were included in the analysis to evaluate the effect of VR on postoperative delirium [23, 26, 30]. A fixed-effects model was used in the meta-analysis as the model showed no heterogeneity (Q = 0.326, df = 2, I^2^ = 0.0%, Tau^2^ = 0.000). The meta-analysis indicated that VR interventions led to a very small reduction in postoperative delirium compared to the control group; however, this effect was not statistically significant (Hedges' g = − 0.102, 95% CI = − 0.298 to 0.094, p = 0.307). Publication bias was not detected, as confirmed by Kendall’s tau without continuity correction (p = 0.60151) and with continuity correction (p = 1.000), as well as Egger’s test (p = 0.45242) (Fig. 5b).

## Discussion

This study is one of the first comprehensive meta-analyses to examine the effects of VR on preoperative, intraoperative, and postoperative anxiety. The analysis evaluated the impact of VR on anxiety, pain, and delirium in pediatric surgical patients across different perioperative phases.

### Effect of VR in the preoperative period

The findings of this study indicate that VR interventions significantly reduced preoperative anxiety. This result is consistent with the meta-analysis conducted by Koo et al. (2020), which reported a significant reduction in anxiety levels among pediatric patients following VR interventions, while no substantial effect was observed in adult populations [36]. Similarly, a systematic narrative review by Antonovics et al. (2024), titled "Use of Virtual Reality in Children in a Broad Range of Medical Settings: A Systematic Narrative Review of Recent Meta-Analyses," also highlighted VR’s potential in mitigating preoperative anxiety in pediatric populations [37]. The present study corroborates these findings, supporting the efficacy of VR as a non-pharmacological approach to anxiety management in pediatric surgical patients. Despite these promising results, the high heterogeneity observed in the model (I^2^ = 55.4%) suggests that variations in VR content, duration of exposure, and methods of anxiety assessment across the included studies may have influenced the outcomes. [21–25, 28–30, 32, 33] In particular, the diversity in VR scenarios and levels of immersion is an essential factor that may have contributed to differences in effect sizes. The variability in content complexity, interactivity, and patient engagement could explain the inconsistencies observed among studies. Addressing these differences through standardization of VR content, intervention protocols, and anxiety assessment tools in future research may enhance the reliability and generalizability of findings.

In our analysis examining the effect of VR on preoperative anxiety, publication bias was detected (Kendall’s tau p = 0.02535; Egger’s test p = 0.01621). Publication bias typically arises when studies with positive results are more frequently published, whereas studies with negative or statistically non-significant findings are less likely to be reported. The presence of publication bias suggests that the observed effects may appear stronger than they actually are, potentially overestimating the true impact of VR interventions on preoperative anxiety.

### Effect of VR in the ıntraoperative period

Meta-analysis results indicate that virtual reality (VR) interventions significantly reduce intraoperative anxiety at a moderate level. For pediatric patients, this period represents not only the beginning of surgery but also a psychologically sensitive phase marked by separation from parents and exposure to an unfamiliar, often intimidating environment. During this transition, anxiety levels can rise considerably. VR offers an immersive and engaging experience that redirects the child’s attention away from the surgical surroundings, providing both distraction and emotional relief. Previous meta-analyses support these findings. Eijlers et al. (2019) demonstrated that VR effectively reduced anxiety across a variety of medical procedures, including oncology, venous access, burn care, and dental treatments [13]. Similarly, Simonetti et al. reported reduced anxiety during the perioperative period in children undergoing elective surgery, although intraoperative effects were not explicitly analyzed [3]. The present meta-analysis, however, provides more targeted evidence by specifically evaluating VR’s role in reducing anxiety during the intraoperative phase. These results suggest that VR can help pediatric patients better manage the emotional challenges of anesthesia induction and intraoperative transitions. As a non-pharmacological intervention, VR shows promise in improving psychological comfort during surgery. Further research is warranted to refine the optimal content, interaction levels, and timing of VR application to maximize its anxiolytic effects in pediatric surgical settings.

### Effect of VR in the postoperative period

Meta-analysis results suggest that while VR interventions significantly reduced postoperative pain, this effect was not statistically significant. This finding is consistent with the meta-analysis conducted by Simonetti et al. (2022), which also found no significant effect of VR on postoperative pain management. In contrast, the meta-analysis by Eijlers et al. (2019) reported that VR effectively reduced pain in pediatric patients undergoing medical procedures. The same study found that VR was particularly effective in reducing pain during burn treatments, whereas its impact on pain reduction in oncological procedures was not statistically significant [13]. Similarly, the meta-analysis by Lluesma-Vidal et al. (2022) demonstrated that VR served as an effective distraction method for reducing pain and fear in pediatric patients during needle-related procedures [38]. Although several studies indicate that VR can be an effective non-pharmacological intervention for pain reduction in children, the methodological limitations and heterogeneity of the included studies restrict the generalizability of these findings [39–41]. Many of the analyzed studies had small sample sizes and inconsistent methodologies, contributing to variations in reported outcomes. Therefore, future research with larger sample sizes and more robust study designs is necessary to better determine the effectiveness of VR in postoperative pain management and to optimize its clinical implementation in pediatric surgical patients..

Meta-analysis results indicate that VR had no significant effect on postoperative delirium (Hedges' g = -0.103, 95% CI = -0.300 to 0.094, p = 0.071). Similarly, the meta-analysis conducted by Simonetti et al. (2022) found no statistically significant impact of VR on postoperative delirium. Due to the limited number of studies in this area, further large-scale and long-term follow-up research is needed to better understand the potential effects of VR on delirium. Additionally, future studies should explore the specific components of delirium—such as cognitive impairment, agitation, and disorientation—to provide a more detailed assessment of how VR may influence these different aspects.

This meta-analysis presents several methodological limitations. First, the inclusion of pediatric patients across a wide age range introduces heterogeneity related to cognitive development, pain perception, and anxiety regulation, thereby limiting the generalizability of the findings. Second, the use of different measurement tools with varying psychometric properties to assess anxiety, pain, and delirium may reduce measurement equivalence and increase the risk of bias in pooled effect estimates. Moreover, substantial heterogeneity was observed among the included studies in terms of VR content, methodological approaches, and sample characteristics, which complicates the interpretation of the results. The presence of publication bias in some studies further suggests that the findings should be interpreted with caution. Future research should prioritize large-scale, multicenter randomized controlled trials comparing different types of VR content to enhance the reliability and clinical applicability of the results.

## Conclusion

This meta-analysis demonstrates that VR is an effective intervention for reducing anxiety in pediatric surgical patients during the preoperative, intraoperative, and postoperative periods. However, considering the heterogeneity and methodological differences among the included studies, further randomized controlled trials (RCTs) are necessary to strengthen these findings. To support the widespread use of VR in surgical procedures and enhance its integration into clinical practice, high-quality, large-scale RCTs are required. Future studies should focus on standardizing VR content, optimizing intervention protocols, and assessing long-term effects to establish VR as a reliable non-pharmacological tool in pediatric perioperative care.

### Practical ımplication

With its ability to effectively reduce anxiety before and in the midst of surgery, VR offers an appealing option as a replacement for traditional anxiety-reduction measures based on pharmacologic intervention. Application of VR to clinical settings has the potential to provide a fuller approach to pediatric care, reduce the use of drugs, and reduce side effects, especially among children who are possibly more drug-sensitive. In an effort to increase the efficiency of VR interventions, future practice in clinical settings should focus on optimizing VR content across different populations of patients to ensure maximum levels of engagement and immersion. Healthcare professionals should be trained to adapt VR experiences according to the needs and preferences of pediatric patients for them to attain optimal comfort and distraction during procedures. Through increasing the utilization of VR in these disciplines, health professionals are able to maximize patient outcomes in addition to maximizing the patient experience.

## Data Availability

No datasets were generated or analysed during the current study.
